# Indole-3-acetic acid-mediated self-rescue in *Bacillus licheniformis* against *Saccharomyces cerevisiae* stress

**DOI:** 10.1093/ismeco/ycag064

**Published:** 2026-03-18

**Authors:** Lei Xu, Chen Ling, Yun Wang, Jiangnan Han, Shihao Liu, Shibo Ban, Qun Wu

**Affiliations:** Key Laboratory of Industrial Biotechnology of Ministry of Education, State Key Laboratory of Food Science and Resources, School of Biotechnology, Jiangnan University, Wuxi 214122, China; Key Laboratory of Industrial Biotechnology of Ministry of Education, State Key Laboratory of Food Science and Resources, School of Biotechnology, Jiangnan University, Wuxi 214122, China; Key Laboratory of Industrial Biotechnology of Ministry of Education, State Key Laboratory of Food Science and Resources, School of Biotechnology, Jiangnan University, Wuxi 214122, China; Key Laboratory of Industrial Biotechnology of Ministry of Education, State Key Laboratory of Food Science and Resources, School of Biotechnology, Jiangnan University, Wuxi 214122, China; Key Laboratory of Industrial Biotechnology of Ministry of Education, State Key Laboratory of Food Science and Resources, School of Biotechnology, Jiangnan University, Wuxi 214122, China; Key Laboratory of Industrial Biotechnology of Ministry of Education, State Key Laboratory of Food Science and Resources, School of Biotechnology, Jiangnan University, Wuxi 214122, China; Key Laboratory of Industrial Biotechnology of Ministry of Education, State Key Laboratory of Food Science and Resources, School of Biotechnology, Jiangnan University, Wuxi 214122, China

**Keywords:** biofilm, coculture, indole-3-acetic acid, microbial interaction

## Abstract

Microorganisms have evolved complex metabolic adaptations to environmental stresses in intricate microbial communities. However, the adaptive strategies of bacteria against the competitive stress from fungi are still poorly understood. Here, we reported an adaptive strategy adopted by *Bacillus licheniformis* in response to *Saccharomyces cerevisiae* stress. The growth of *B. licheniformis* in the presence of *S. cerevisiae* was reduced at 24 h, followed by a complete recovery after 48 h, coupled with a full-process reduction in biofilm formation. Meantime, genes involved in carbohydrate metabolism were significantly upregulated, and those involved in biofilm formation were significantly downregulated in *B. licheniformis* at 24 h. When *B. licheniformis* was cultured in *S. cerevisiae* conditioned medium, differentially upregulated metabolites after 24 h of incubation were primarily enriched in tryptophan metabolism pathway, with significant accumulation of indole-3-acetic acid (IAA) and its precursors. Supplementation with IAA suppressed biofilm formation in a concentration-dependent manner and promoted biomass recovery at later fermentation stages, as well as upregulation of genes involved in carbohydrate metabolism and downregulation of genes involved in biofilm formation at 24 h. This study revealed that IAA acted as a bacterial signaling molecule via reducing biofilm formation to promote the growth recovery against the stress from fungi.

## Introduction

In natural niches, microorganisms often face various environmental stresses [1, 2]. Under environmental stress, bacterial survival is governed by sophisticated adaptive strategies that ultimately determine their population fitness within microbial communities [3]. These adaptive responses engage global regulatory networks to coordinate the growth-survival trade-off under environmental stresses [4]. Thus, elucidating how bacteria integrate growth and survival strategies under such stresses would not only reveal the fundamental principles of microbial adaptation but also illuminate the chemical and ecological processes that shape species coexistence in dynamic environments.

Microorganisms have developed intricate adaptive strategies to cope with biotic and abiotic stresses [5]. For example, *Rhodanobacter* gains adaptation by losing its flagella and enhancing biofilm formation under low pH and aluminum stress [6]. *Rhodococcus ruber* promotes the production of vitamin B_12_ under high salinity stress, a strategy that aids in biofilm formation and cofactor synthesis, thereby enhancing osmotolerance [7]. These adaptive mechanisms are coordinated through regulated signaling networks, enabling microorganisms to sense and respond to environmental stresses. However, it remains unclear how bacteria reallocate metabolic resources to sustain fitness under competitive stress.

*Bacillus* is widely distributed in soil, plant, marine, and food fermentation systems, where it plays an important role in shaping microbial community dynamics [8]. *Bacillus* can thrive in diverse ecological settings by deploying multiple survival strategies, including sporulation, biofilm formation, and antimicrobial compound secretion [9]. These adaptations allow *Bacillus* to thrive under diverse environmental stresses such as nutrient limitation [10], ethanol stress [11], and microbial competition [12]. In microbial communities, competitive and cooperative interactions with other species often change *Bacillus* physiology [13]. Previous studies have shown that *Saccharomyces cerevisiae* inhibits *Bacillus* growth through ethanol production and environmental acidification [14]. However, *Bacillus* and *S. cerevisiae* coexist in various food fermentations [15–17]. Such coexistence–competition scenarios provide an ecologically relevant model to study how bacteria survive under fungal stress.

This study aimed to explore the adaptation mechanisms of *Bacillus licheniformis,* a versatile species with important roles in nature and food fermentation systems [18], against *S. cerevisiae* stress. We first revealed the adaptation pattern, including cell growth dynamics and biofilm formation of *B. licheniformis* against *S. cerevisiae* stress, using coculture and conditioned-culture. Then we characterized the variation of gene transcription and metabolites production to identify candidate metabolites associated with the adaptive response in *B. licheniformis* using transcriptomic and metabolomic analysis. Finally, we verified the role of candidate metabolites in regulating the adaptive response using supplementary experiments and transcriptomic analysis. This study expands our understanding of the mechanisms by which microbial metabolites function as signaling molecules, with a particular focus on their multifaceted roles in bacterial-fungal interactions within complex environments.

## Materials and methods

### Strains

*Saccharomyces cerevisiae* MT1 (China Center for Type Culture Collection, accession number: CCTCC M2014463) and *B. licheniformis* MT6 (China General Microbiological Culture Collection Center, accession number: CGMCC3963) were previously isolated from Chinese liquor fermentation [14].

### Monoculture and coculture experiments

Monoculture and coculture experiments were conducted using chemically defined medium [19] to effectively support the growth of both *B. licheniformis* and *S. cerevisiae*. Three experimental sets were designed: (i) monoculture vs coculture growth comparisons; (ii) supplementation with potential indole-3-acetic acid (IAA) precursors: tryptophan, tryptamine, indole-3-acetamide (IAM); and (iii) supplementation with tryptamine, indole-3-acetamide, or IAA at different concentrations and time points. A complete description of the protocol is given in Supplementary Methods S1.

### Culture experiments with conditioned medium

Based on the growth curves, both *B. licheniformis* and *S. cerevisiae* reached the late stage of logarithmic phase at 12 h in chemically defined medium, and growth suppression of *B. licheniformis* in coculture started at 12 h. Therefore, conditioned media were prepared from 12 h cultures of each species. *B. licheniformis* was inoculated into conditioned medium of *S. cerevisiae* to explore the effects of metabolites of *S. cerevisiae* on *B. licheniformis. S. cerevisiae* was inoculated into conditioned medium of *B. licheniformis* to explore the effects of metabolites of *B. licheniformis* on *S. cerevisiae*. A complete description of the protocol is given in Supplementary Methods S2.

### DNA extraction and quantitative polymerase chain reaction analyses

Genomic DNA was extracted using rapid bacterial genomic DNA isolation kit (Sangon Biotech, Shanghai, China) for *B. licheniformis* and rapid yeast genomic DNA isolation kit for *S. cerevisiae* in monoculture and coculture. All the operations followed the manufacturer’s instructions. Genomic DNA was used as the template to amplify *B. licheniformis* using primers Bl-F and Bl-R [20], *S. cerevisiae* using primers SC-F and SC-R [21]. Primer sequences were shown in Table S1. Each reaction step followed the previous description [22] on the StepOnePlus instrument (Applied Biosystems, Foster City, CA).

### Field emission scanning electron microscope

Monoculture and coculture samples at 24-hour time point were harvested by centrifugation at 8000 rpm for 5 min at 4°C. Cells were fixed with 2.5% (v/v) glutaraldehyde at 4°C overnight. After fixation, the samples were washed three times with 0.1 M phosphate buffer saline (PBS) and dehydrated in an ethanol gradient (30%, 50%, 70%, 90%, and 100%, v/v). The dehydrated samples were dried at room temperature. Finally, samples were coated with gold and observed with a field emission scanning electron microscope (FEI Quanta-200, Hitachi, Japan).

### Biofilm quantification

We used two methods to quantify biofilm. Strains were incubated at 30°C and 100 rpm for 48 h in 24 deep-well plates. For dry weight method, all cell cultures were collected, and the biofilm attached to the chamber was rinsed with 0.1 M PBS. Biofilm was filtered through a 0.45 μm filter membrane, which had been pre-dried and weighed, and then dried at 105°C until the weight did not change to obtain the dry weight. For the crystal violet staining method, the procedures were performed as previously described [23].

### RNA extraction and reverse transcription quantitative polymerase chain reaction analyses

Total RNA was extracted from the cell cultures using TRIzol® Reagent according to the manufacturer’s instructions. The cDNA was synthesized using HiScript IV All-in-One Ultra RT SuperMix for quantitative polymerase chain reaction (qPCR) (Vazyme) in a 20 μl reaction volume with 1 μg of total RNA. Nine biofilm-related genes were selected to determine the accuracy of the transcriptome using reverse transcription quantitative polymerase chain reaction (RT-qPCR) analyses. The reactions were performed as previously described [22] using primers listed in Supplementary Table S1, and the *recA* gene was selected as the housekeeping gene. The relative gene expression was calculated using the 2 ^−ΔΔCt^ method.

### RNA sequencing and transcriptome analysis

RNA sequencing was performed for both *B. licheniformis* and *S. cerevisiae*. Differential expression analysis was performed using DESeq2. Genes with |log_2_(fold change, FC) | ≥1 and *P*_adj_ < 0.05 were considered to be significantly differentially expressed genes (DEGs). DEGs were annotated using eggNOG-mapper [24] (v. 2.1.12) against the eggNOG database [25] (v. 5.0.2). In addition, Kyoto Encyclopedia of Genes and Genomes (KEGG) [26] functional enrichment analysis was performed to identify which DEGs were significantly enriched in metabolic pathways at Bonferroni-corrected *P*-value <0.05 compared with the background. A complete description of the protocol is given in Supplementary Methods S3.

### Metabolites extraction and metabolomics analysis

A 1 ml of cultured solution collected at 24 h was mixed with 400 μl methanol solution, and vortexed. The mixture was centrifuged for 10 min at 12000 rpm and 4°C. Then, the sample was concentrated and dried. The samples were redissolved in 150 μl of 2-chloro-L-phenylalanine (4 μg/ml) solution prepared in 80% methanol, followed by filtration through a 0.22 μm membrane filter. The filtrate was transferred to a vial for liquid chromatography–mass spectrometry (LC–MS) analysis. Metabolites with VIP >1 and *P*_adj_ <0.05 were considered to be differentially abundant metabolites, indicating significant differences in relative abundance between groups. A complete description of the protocol is given in Supplementary Methods S4.

### Quantification of indole-3-acetic acid, indole-3-acetamide, and tryptamine

To confirm whether IAM and tryptamine were precursors of IAA and to determine the IAA production of *B. licheniformis* in monocultures and cocultures, we quantified IAA, IAM, and tryptamine by ultra performance liquid chromatography–mass spectrometry/mass spectrometry (UPLC-MS/MS). A complete description of the protocol is given in Supplementary Methods S5.

### Statistics

Data were analyzed using SPSS Statistics 27 (IBM, Armonk, NY, USA). For comparisons between two groups, the differences between means were analyzed using the *t-*test. For multiple-group comparisons, Fisher’s least significant difference (LSD) test was performed for pairwise comparisons. The linearity of the standard curve in qPCR experiment was assessed using the linear fitting function in Origin (OriginLab, Northampton, MA, USA).

## Results

### Growth recovery in *Bacillus licheniformis* under *Saccharomyces cerevisiae* stress

**Figure 1:**
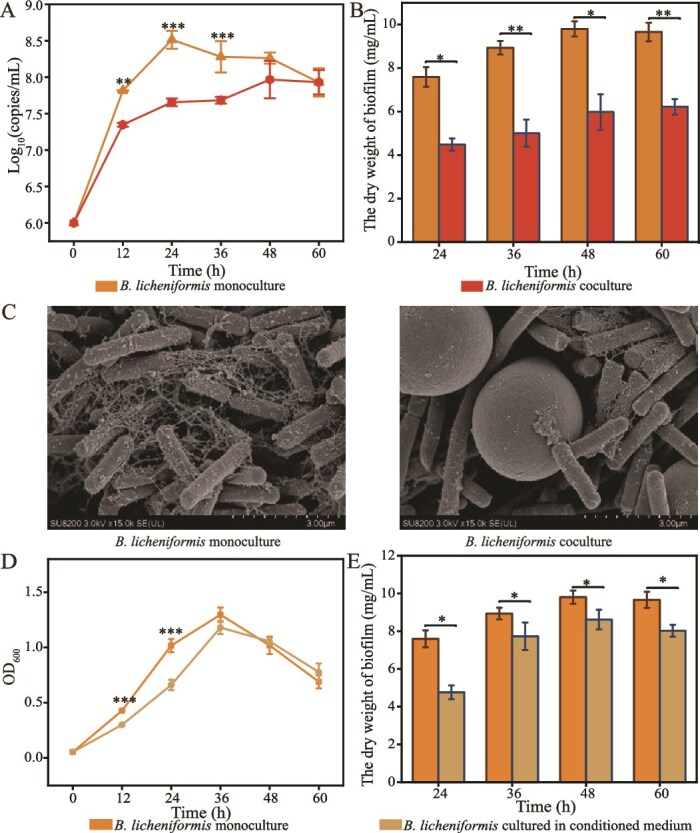
Variations in biomass and biofilm of *B. licheniformis* in the presence of *S. cerevisiae*. (A) The biomass of *B. licheniformis* in monoculture and coculture. (B) Dry weight of biofilm of *B. licheniformis* in monoculture and coculture. (C) Cell morphology of *B. licheniformis* in monoculture and coculture at 24 h. (D) The biomass of *B. licheniformis* in monoculture and conditioned culture of *S. cerevisiae*. (E) Dry weight of biofilm of *B. licheniformis* in monoculture and conditioned culture of *S. cerevisiae*. Significance: ^*^P <0.05, ^**^P <0.01, ^***^P <0.001.

The dynamic profile of *B. licheniformis* was determined in the presence of *S. cerevisiae* along a long fermentation period. The growth of *B. licheniformis* significantly decreased at the beginning and the middle of the fermentation period (0 ~ 36 h), and biomass decreased from 8.51 ± 0.12 to 7.66 ± 0.15 log_10_(copies/ml) at 24 h, compared with that in the monoculture, suggesting *B. licheniformis* was under the physiological stress of *S. cerevisiae*. However, *B. licheniformis* still kept growing, and the growth in the late period of fermentation (48 ~ 60 h) was equivalent to that without *S. cerevisiae*, indicating growth recovery in *B. licheniformis* (Fig. 1A). In contrast, the biomass of *S. cerevisiae* showed no significant difference in the presence of *B. licheniformis* before 36 h, but it decreased quickly in the presence of *B. licheniformis* at 48 h (Fig. S1).

*B. licheniformis* produced biofilm during fermentation. However, biofilm formation significantly decreased throughout the fermentation period in the presence of *S. cerevisiae* (Fig. 1B), which was supported by reduced flocculent biofilm observed at 24 h (Fig. 1C, Fig. S2).

To further investigate whether the variation in *B. licheniformis* was mediated by cell–cell contact or metabolic effects, we used conditioned medium to investigate the influence of *S. cerevisiae* on *B. licheniformis*. The biomass of *B. licheniformis* reduced at the beginning and the middle of the fermentation period in the conditioned medium of *S. cerevisiae*, with a 35.05% decrease compared to that with chemically defined media in monoculture at 24 h (*P* <0.01, *t*-test). However, no significant difference was observed after 36 h (Fig. 1D). In addition, the formation of biofilm also decreased during the whole fermentation of *B. licheniformis* in the conditioned medium (*P* <0.05, *t*-test, Fig. 1E). The biomass of *S. cerevisiae* was significantly higher when cultured in the conditioned medium of *B. licheniformis* at 48 h, compared to monoculture (*P* <0.01, *t*-test, Fig. S3). These findings suggested that *S. cerevisiae* imposed metabolic stress on *B. licheniformis*, and *B. licheniformis* adapted to *S. cerevisiae* stress in the late period of fermentation.

### Increase of transcriptional activity of *Bacillus licheniformis* under *Saccharomyces cerevisiae* stress

**Figure 2:**
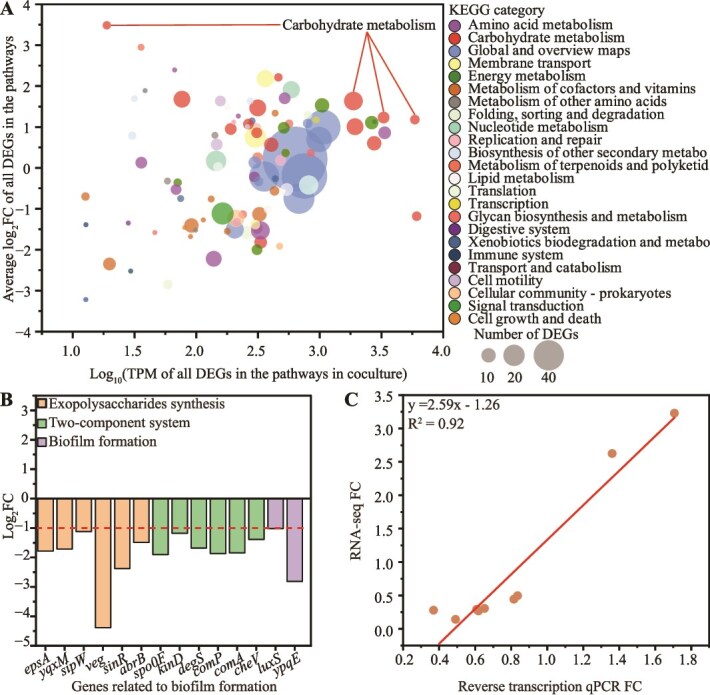
Variation of the transcriptomic profile of *B. licheniformis* in the presence of *S. cerevisiae*. (A) KEGG pathway summary of DEGs at the pathway level grouped by KEGG categories. The x-axis shows the expression intensity of each pathway; the y-axis shows the average fold change (FC). Bubble size indicates the number of DEGs. (B) Fold changes in genes related to biofilm formation. (C) Validation of RNA-seq by reverse transcription qPCR for *epsA, yqxM, Spo0F, kinD, comP, comA, luxS*, and *ypqE* (*y* = 2.59x – 1.26, *R*^2^ = 0.92). TPM, transcripts per million; FC, fold change.

To uncover the molecular mechanisms of growth recovery in *B. licheniformis*, we performed RNA sequencing of *B. licheniformis* in monoculture and coculture with *S. cerevisiae* at 24 h. A total of 866 (|log_2_FC| ≥ 1 and *P*adj <0.05, 311 upregulated and 555 downregulated) DEGs were identified in *B. licheniformis* in the presence of *S. cerevisiae*, compared with that in monoculture (Fig. S4). DEGs were annotated according to KEGG categories (Fig. 2A). DEGs involved in carbohydrate metabolism and nucleotide metabolism categories were biased toward upregulation (average log₂FC of all DEGs in the pathways ≥1 with >20 DEGs per category, Fig. S5). Within the carbohydrate metabolism category, DEGs involved in starch and sucrose metabolism, fructose and mannose metabolism, and glycolysis/gluconeogenesis pathways were predominantly upregulated (average log₂FC of all DEGs in the pathways ≥1, Fig. S5) together with high expression level in coculture [log₁₀(transcripts per million of all DEGs in the pathway) ≥3)]. Conversely, DEGs involved in metabolism of cofactors and vitamins and signal transduction categories were biased toward downregulation (average log₂FC of all DEGs in the pathways ≤ − 1 with >20 DEGs per category). Within signal transduction category, DEGs involved in the two-component system pathway were predominantly downregulated (average log₂FC ≤ −1). These results indicated that although the cell growth was inhibited, the transcription remained active for preparing the growth recovery.

Because the two-component system pathway is implicated in biofilm formation in *Bacillus*, we further examined biofilm-related genes transcription. DEGs involved in exopolysaccharide synthesis (*epsA, yqxM, sipW, veg, sinR,* and *abrB*), two-component systems (*spo0F, kinD, degS, comP,* and *comA*), and biofilm formation (*luxS* and *ypqE*) were significantly downregulated in the coculture (Fig. 2B). These findings suggested that *B. licheniformis* in the presence of *S. cerevisiae* may suppress biofilm formation by inhibiting exopolysaccharide biosynthesis, potentially involving in modulation of biofilm-related signaling pathways.

To validate these RNA-seq findings, we performed RT-qPCR on a subset of nine DEGs associated with biofilm formation (Fig. S6). The RT-qPCR results were consistent with the RNA-seq data (*R*^2^ = 0.92), further confirming the reliability of the observed transcriptional changes (Fig. 2C).

### Candidate metabolites mediated the growth recovery in *B. licheniformis* under *Saccharomyces cerevisiae* stress

To determine whether there are metabolites mediating the growth recovery in *B. licheniformis*, we performed nontargeted metabolomics to compare metabolites in the culture of *B. licheniformis* in the conditioned medium at 24 h and in the original conditioned medium prepared from *S. cerevisiae*. Principal component analysis (PCA) demonstrated that the metabolic profiles of *B. licheniformis* were markedly different between the two culture systems (Fig. S7).

Compared to the original conditioned medium, 70 differentially abundant metabolites (VIP >1, *P* <0.05) were identified in *S. cerevisiae* cultured in conditioned medium of *B. licheniformis* at 24 h (Fig. S8). Of these, the relative abundances of 27 metabolites significantly increased, and those of 43 significantly decreased. The relative abundances of organic acids, such as α-ketoisovaleric acid and tartaric acid, in *S. cerevisiae* cultured in the conditioned medium of *B. licheniformis* at 24 h were higher than those in the original conditioned medium. These results suggested that *S. cerevisiae* may impose acid stress.

**Figure 3:**
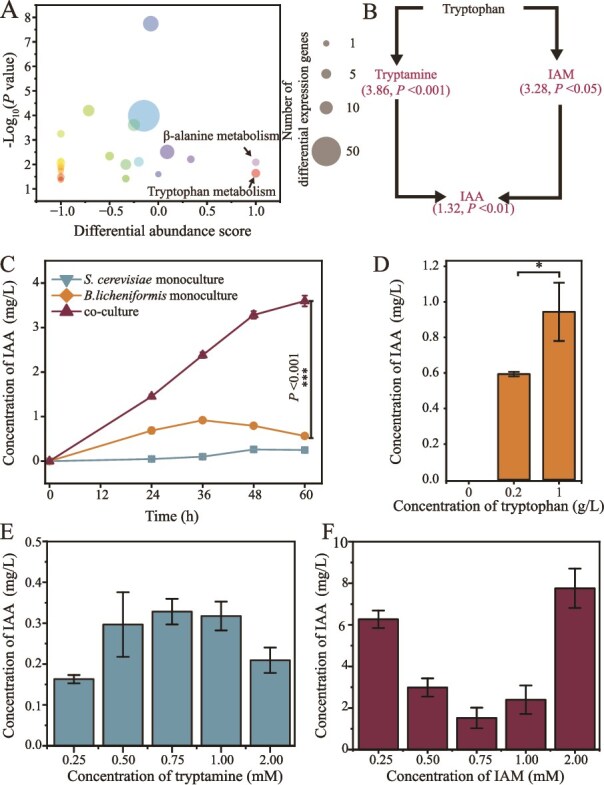
Candidate metabolites of *B. licheniformis* involving adaptation to *S. cerevisiae* stress and its synthesis pathway analysis. (A) Differential abundance scores of metabolites after enrichment. Differential abundance score = (number of upregulated metabolites – number of downregulated metabolites)/total number of differentially abundant metabolites in the pathway. (B) Proposed IAA biosynthesis pathway. Red words highlight significantly increased metabolites. (C) Concentration of IAA in mono- and coculture of *B. licheniformis*. (D–F) Effect of tryptophan (D), tryptamine (E), and IAM (F) on IAA concentration in *B. licheniformis* monoculture with tryptophan-depleted medium. The concentration of IAA was detected at 48 h in the fermentation.

Compared to the original conditioned medium, 81 differentially abundant metabolites were identified in *B. licheniformis* cultured in conditioned medium at 24 h (Fig. S9). Of these, the relative abundances of 38 metabolites significantly increased, and those of 43 significantly decreased. The KEGG pathway enrichment analysis revealed that differentially abundant metabolites were primarily enriched in β-alanine metabolism and tryptophan metabolism pathways, and the differentially abundant metabolites in these two pathways significantly increased (Fig. 3A). Notably, DEGs involved in tryptophan metabolism pathway were also predominantly upregulated in transcription (average log₂FC of all DEGs in the pathways = 1.27, Supplementary Table S2) in *B. licheniformis* in the coculture compared with that in monoculture, confirming the role of tryptophan metabolism pathway in growth recovery of *B. licheniformis*. In the tryptophan metabolism pathway, significantly increased metabolites were identified to be involved in IAA biosynthesis. The relative abundances of IAM, tryptamine, and IAA in *B. licheniformis* in the conditioned culture system were 3.28, 3.86, and 1.32-fold higher than those in the original conditioned medium, respectively (Fig. 3B). To verify the result, we determined the dynamics of IAA concentration in monoculture and coculture of *B. licheniformis* and *S. cerevisiae* during the fermentation. The final concentration of IAA reached 3.60 ± 0.12 mg/l in the coculture, significantly higher than that in the monoculture of both *B. licheniformis* and *S. cerevisiae* (*P* <0.001, Fig. 3C), indicating biosynthesis of IAA increased in *B. licheniformis* in the presence of *S. cerevisiae*.

To verify whether IAM and tryptamine were involved in IAA biosynthesis, we separately added different concentrations of tryptophan, tryptamine, and IAM in tryptophan-depleted chemically defined medium and determined the concentrations of IAA produced by *B. licheniformis*. No IAA was detected in tryptophan-depleted chemically defined medium, but IAA increased to 0.59 ± 0.01 mg/l and 0.94 ± 0.16 mg/l when 0.2 g/l and 1 g/l of tryptophan were separately added to this medium in the monoculture of *B. licheniformis* (Fig. 3D), indicating the essential role of tryptophan in IAA biosynthesis. In addition, IAA can also be produced by *B. licheniformis* in the tryptophan-depleted chemically defined medium, when tryptamine and IAM were separately added at concentrations ranging from 0.25 to 2 mM (Fig. 3E and F), indicating that tryptamine and IAM were also precursors of IAA in *B. licheniformis*. In addition, the concentration of IAA was much higher with the addition of IAM than with tryptamine, indicating IAM might be a more important precursor of IAA. These results indicated that *B. licheniformis* enhanced the biosynthesis of IAA, which might be the final metabolite related to growth recovery in *B. licheniformis* under *S. cerevisiae* stress.

### Indole-3-acetic acid mediated the decrease of biofilm formation and the increase of cell growth in *Bacillus licheniformis*

**Figure 4:**
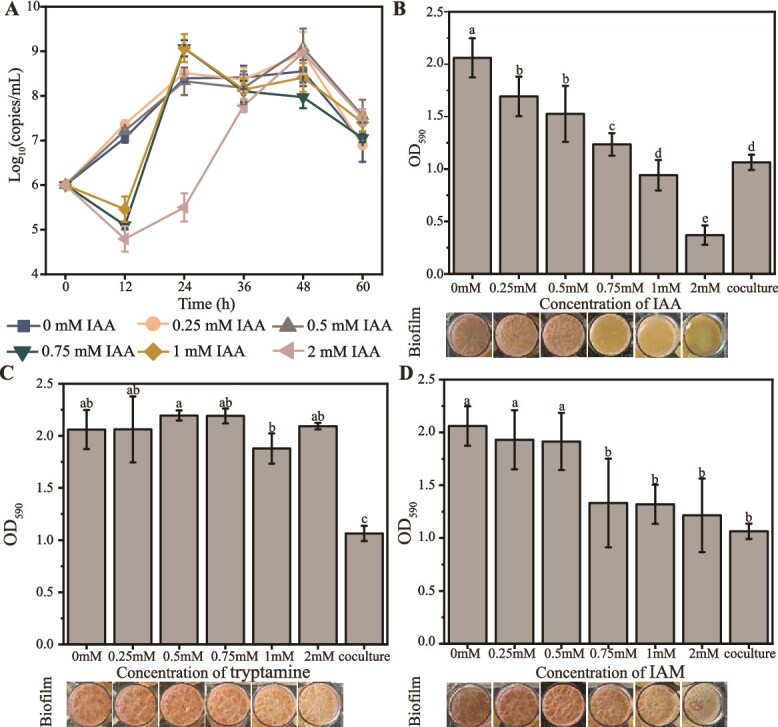
Effects of IAA on the growth and biofilm formation of *B. licheniformis*. (A) Growth curves of *B. licheniformis* cultured in chemically defined medium supplemented with different concentrations of IAA. (B–D) Changes in biofilm formation of *B. licheniformis* with the addition of different concentrations of IAA (B), tryptamine (C), and indole-3-acetamide (IAM) (D) to the chemically defined medium. Different letters above the bar indicate a significant difference (Fisher LSD, *P* <0.05) among groups, and the same letter indicates no significant difference. Biofilm was quantified at 24 h incubation in 24-well plates by crystal violet staining (OD_590_).

We hypothesized that IAA played a crucial role in the growth recovery of *B. licheniformis* under *S. cerevisiae* stress. To verify the hypothesis, we determined the effect of IAA on the growth and biofilm formation of *B. licheniformis* by adding different concentrations of IAA to chemically defined medium of *B. licheniformis* in monoculture. When the supplementary concentration was 0.25 mM, biomass of *B. licheniformis* was significantly higher than that of monoculture at 12 h (*P* <0.05, *t*-test), but no significant differences were observed at 24 h and thereafter. When the supplementary concentration was 0.5 mM, no significant difference was detected at any time point. When the supplementary concentrations were 0.75 mM and 1 mM, growth was markedly inhibited at 12 h (*P* <0.001 and *P* <0.01, *t*-test), but significantly promoted at 24 h (*P* <0.01 and *P* <0.05, *t*-test), and no differences were observed at 36 h and thereafter. When the supplementary concentration was 2 mM, IAA suppressed growth at 12 h (*P* <0.001, *t*-test), 24 h (*P* <0.001, *t*-test), and 36 h (*P* <0.05, *t*-test), but no differences were observed at 48 h and 60 h (Fig. 4A). These results demonstrated that the concentration of supplemented IAA strongly influenced the growth dynamics of *B. licheniformis*. Overall, low concentrations ($\le$0.5 mM) of IAA had little effect on the growth of *B. licheniformis*, except that 0.25 mM IAA slightly promoted growth at 12 h. In contrast, when the concentration was 0.75 mM and 1 mM, IAA inhibited the growth of *B. licheniformis* at 12 h, followed by a promoting effect at 24 h, and finally exhibited no significant differences at 36 h and thereafter.

Given the biofilm inhibition of *B. licheniformis* under *S. cerevisiae* stress, we further investigated whether IAA regulated the biofilm formation of *B. licheniformis*. The biofilm formation gradually decreased with the increase of IAA concentration from 0.25 mM to 2 mM (Pearson’s *R* = −0.988, *P* <0.001) (Fig. 4B). Moreover, we tested the effect of other metabolites in the tryptophan metabolism pathway by separately adding IAM and tryptamine to the chemically defined medium. The results showed that the addition of tryptamine did not significantly influence biofilm formation (Fig. 4C), but IAM significantly decreased biofilm production at the concentration of 0.75 mM, 1 mM, and 2 mM (Fig. 4D). These findings supported the hypothesis that IAA, rather than its precursors, is the key modulator of cell growth and biofilm formation in *B. licheniformis*. These results suggested a hypothesis that *B. licheniformis* might enhance IAA production via the tryptophan pathway to reduce biofilm formation and improve its growth under the stress of *S. cerevisiae*.

In addition, to check the actual producer of IAA in the coculture, we examined the variation in metabolites of *S. cerevisiae* in conditioned-culture compared with that in the original conditioned medium prepared from *B. licheniformis*. The relative abundances of IAA and its precursors showed no significant differences between the *S. cerevisiae* conditioned culture and the original conditioned medium. Moreover, we examined the variation in transcription of *S. cerevisiae* in coculture compared with that in the monoculture. DEGs involved in IAA biosynthesis (EC:3.5.5.1, log_2_FC = −1.84 and EC:1.2.1.3, log_2_FC = −4.06) were downregulated in coculture (Supplementary Table S3). These results indicated that the elevated IAA observed in coculture was not derived from *S. cerevisiae* but rather produced by *B. licheniformis* against *S. cerevisiae* stress.

### Indole-3-acetic acid as a signaling molecule in *Bacillus licheniformis*

**Figure 5:**
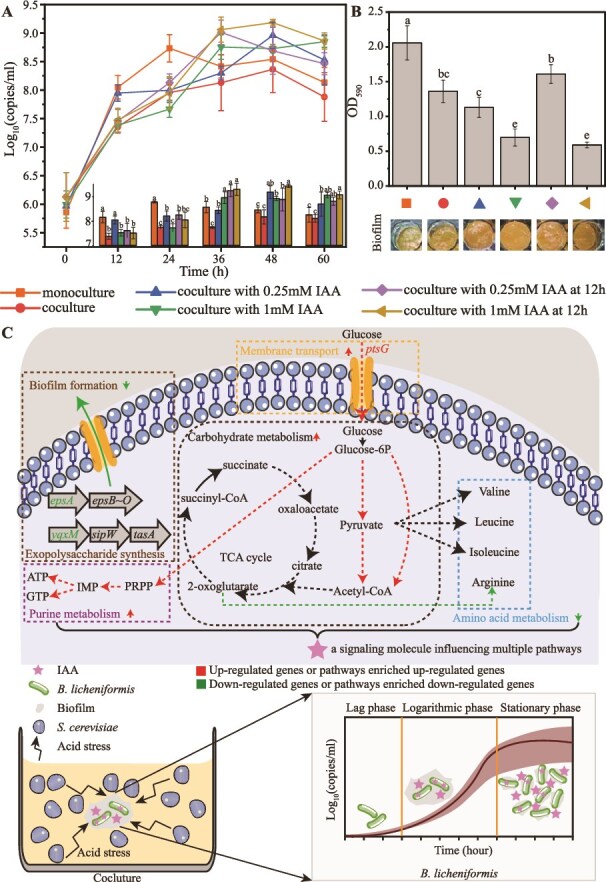
The mechanism of IAA as a signaling molecule in the interaction between *B. licheniformis* and *S. cerevisiae*. (A) Growth curve of *B. licheniformis* in monoculture and coculture with the addition of different concentrations of IAA at different times in chemically defined medium. The inset bar chart summarizes the corresponding values at each time point to facilitate between-group comparisons. The bar chart shows the differences between the different groups. Different letters above the bar indicate a significant difference (Fisher LSD, *P* <0.05) among groups, and the same letter indicates no significant difference. (B) Changes in biofilm formation of *B. licheniformis* in monoculture and coculture with the addition of different concentrations of IAA at different times in chemically defined medium. Biofilm was quantified at 24 h incubation in 24-well plates by crystal violet staining (OD_590_). (C) Schematic illustration depicting the role of IAA as a signaling molecule in regulating gene transcription of *B. licheniformis*. In the upper panel, highlighted categories include membrane transport (orange border), carbohydrate metabolism (black border), nucleotide metabolism (purple border), amino acid metabolism (blue border), and exopolysaccharide synthesis (brown border). Red arrows represent pathways enriched upregulated genes, and green arrows represent pathways enriched downregulated genes. Dashed lines represent multistep reactions, and solid lines indicate direct reactions. Red labels represent upregulated genes, and green labels represent downregulated genes.

To verify the hypothesis that IAA regulates biofilm formation and growth of *B. licheniformis* in the coculture, we examined the effects of IAA on the growth of *B. licheniformis* in the coculture, besides that in the monoculture. Biomass of *B. licheniformis* was significantly promoted at 12 h and thereafter with the supplement of 0.25 mM IAA at the initial of fermentation, compared with that without the addition in the coculture. Biomass of *B. licheniformis* was the same at 12 h and 24 h, but significantly increased at 36 h and thereafter with the supplement of 1 mM IAA at the initial of fermentation, compared with that without the addition in the coculture. It indicated that supplement of 0.25 mM IAA can accelerate the growth recovery of *B. licheniformis* from 48 h to 12 h, and high concentration of IAA (1 mM) can accelerate the growth recovery of *B. licheniformis* from 48 h to 36 h, and both two concentrations of IAA could promote the cell growth at 36 h and thereafter in the coculture (Fig. 5A).

We also analyzed the impact of the supplementary time of IAA on the growth of *B. licheniformis* in the coculture (Fig. 5A). No significant differences were observed at 12 h and 24 h with the supplement of 0.25 mM and 1 mM IAA at 12 h, compared with those without the addition in coculture. However, biomass of *B. licheniformis* was significantly promoted at 36 h and thereafter with the supplement of 0.25 mM and 1 mM IAA at 12 h, compared with that without the addition in the coculture. It indicated that supplement of IAA at 12 h could accelerate the growth recovery of *B. licheniformis* from 48 h to 36 h. In addition, biomass of *B. licheniformis* was higher at 36 h with the supplement of 0.25 mM IAA at 12 h, compared with that with the supplement of 0.25 mM IAA at the initial of fermentation. Importantly, in all IAA-supplemented groups, biomass of *B. licheniformis* at 60 h was significantly higher than those of both the monoculture and coculture without addition. These results demonstrated that the supplementary time of IAA influenced the growth recovery of *B. licheniformis*, and delayed supplementation of IAA would delay its effect, but it would lead to the same growth recovery at the end of fermentation regardless of supplementary time.

We next investigated the effect of IAA on biofilm formation in the coculture. Supplementation with 1 mM IAA significantly suppressed biofilm formation at 24 h compared with the monoculture and untreated coculture. When 0.25 mM IAA was added at the beginning of fermentation, biofilm formation of *B. licheniformis* was lower than that with the supplementation of 0.25 mM IAA at 12 h, and they both exhibited no significant difference compared with the coculture without supplementation. In addition, biofilm formation was equally suppressed with the supplementation of 1 mM IAA at both the beginning and 24 h, and the suppression was much stronger than that in the coculture without supplementation (Fig. 5B). It indicated that IAA could suppress biofilm formation of *B. licheniformis* in the coculture, and the suppression can be influenced by both IAA concentration and the supplementation time. These findings indicated that IAA would contribute to growth recovery not only by promoting biomass accumulation but also by repressing biofilm formation, thereby redirecting resources toward cell proliferation.

To explore the underlying mechanisms, we conducted RNA sequencing of *B. licheniformis* in monoculture of chemically defined medium with or without the addition of 0.25 mM IAA at 24 h. Compared with that without the addition of IAA, a total of 630 DEGs were identified (252 upregulated and 378 downregulated, Fig. S10A). The upregulated DEGs were enriched in phosphotransferase system (PTS), purine metabolism, and fructose and mannose metabolism pathway. Furthermore, the upregulation of carbohydrate metabolism (fructose and mannose metabolism, inositol phosphate metabolism, and glycolysis/gluconeogenesis pathways) suggested that *B. licheniformis* may reprogram its carbon metabolism (Fig. 5C). This metabolic flexibility might explain the observed recovery in *B. licheniformis* biomass when IAA was added in monoculture (Fig. 4A). Notably, the PTS played a crucial role in carbohydrate metabolism and the adaptation of cells to external nutrient changes. The downregulated DEGs were enriched in biosynthesis of amino acids, 2-oxocarboxylic acid metabolism, and C_5_-branched dibasic acid metabolism pathway (Fig. S10B). The significant downregulation of branched-chain amino acid synthesis pathways, coupled with the activation of purine metabolism, provided a metabolic compensation. The downregulation of branched-chain amino acid synthesis reduced the consumption of structural proteins, and the activation of purine metabolism increased nucleotide reserves and supplied energy.

Furthermore, genes associated with biofilm formation, including those involved in exopolysaccharide synthesis (*epsA, yqxM,* and *veg*) and two-component systems (*degU*), were downregulated (Fig. S11). These results indicated that IAA can modulate the synthesis of extracellular polysaccharides in *B. licheniformis*, which might be related to the reduction of biofilm formation.

Overall, we propose a “self-rescue” model as an adaptive strategy in *B. licheniformis*: under acid stress from *S. cerevisiae, B. licheniformis* increases IAA production, suppresses energetically costly biofilm formation, and reallocates resources toward growth, thereby recovering from the initial growth inhibition (Fig. 5C). These findings add valuable insights into the functional diversity of IAA in bacteria.

## Discussion

Over the past decades, studies have revealed a wide range of signaling molecules in *Bacillus*, from the ComX pheromone that regulates sporulation and surfactin biosynthesis [27] to autoinducer-2 and cyclic di-guanylate monophosphate that regulates sporulation, motility, and biofilm formation [28–30], highlighting the complexity of communication in *Bacillus.* Here, we identified IAA, best known as a plant auxin, as a new signaling molecule in *B. licheniformis.* This discovery not only expanded the catalog of signaling molecules in *Bacillus* but also revealed that metabolites with broad biological functions can be employed into bacterial communication.

The identification of IAA as a signaling molecule also broadened our understanding of its functional diversity. IAA has been widely studied in plant-microorganism interactions [31]. In plant-associated bacteria, IAA has frequently been reported to stimulate biofilm formation, enhance root colonization, and support plant growth [32]. Previous studies indicate that IAA stimulates the production of trehalose, lipopolysaccharides, exopolysaccharides, and biofilms, thereby enhancing microbial tolerance to antibiotics and diverse environmental stresses [32–36]. In contrast, our study showed that in *B. licheniformis*, IAA suppressed biofilm formation while facilitating biomass recovery under *S. cerevisiae* stress. This divergence indicated that the regulatory effects of IAA are not universal but vary with the genetic regulatory architecture of the host, and it should be viewed as a versatile signaling molecule whose impact on bacterial physiology varies across biological systems. Understanding the mechanisms by which IAA exerts these effects on metabolism and social behavior therefore represents an important direction for future research.

To verify the contrasting effect of IAA on biofilm formation in this work, we further examined how IAA regulates biofilm formation. Transcriptomic analysis of IAA supplementation in *B. licheniformis* monoculture revealed that genes related to biofilm matrix formation (*epsA* and *yqxM*) and transcription factors (*veg* and *degU*) were consistently downregulated (Fig. S11). *DegU* is a direct activator of *pgsB* transcription involved in *γ*-poly-glutamic acid synthesis [37] and *Veg* proteins are known to promote biofilm formation through the *Spo0A*–*SinR* cascade in *Bacillus* [38]. These results suggested that IAA acted upstream of biofilm formation, interfering with regulatory pathways that govern biofilm formation. One possibility was that IAA influenced *veg* transcription and thereby indirectly modulated SinR activity; another was that it might be sensed through a yet-unidentified receptor system. In either case, IAA represented a new regulatory factor within the *Bacillus* biofilm formation, underscoring the flexibility and complexity of its communication networks.

In addition to repressing biofilm formation, IAA also promoted the growth of *B. licheniformis* in coculture. This dual outcome suggested that IAA influenced resource allocation beyond biofilm regulation alone. Biofilm matrix production is metabolically costly, diverting carbon, nitrogen, and energy from cell growth [39]. IAA might alleviate this burden and redirect resources toward central metabolic pathways that sustain cell growth. Transcriptomic analysis revealed upregulation of genes involved in carbohydrate metabolism (average log₂FC of all DEGs in the pathways ≥1 with >20 DEGs per category) and a concomitant downregulation of genes involved in biofilm formation (Figs S6 and S11). Thus, this metabolite-mediated allocation strategy likely allowed *B. licheniformis* to balance metabolic resources, providing *B. licheniformis* with a competitive advantage under fungal stress.

IAA is generally considered to be endogenously synthesized and functions intracellularly; only a small amount of IAA might be secreted extracellularly [40]. In our supplementation experiments, the concentrations of IAA added to the culture that resulted in significant phenotypic effects were higher than those detected in coculture supernatants. Intracellular accumulation of IAA, which is not reflected in extracellular assays, might account for this discrepancy. Moreover, IAA can be degraded into oxidized derivatives such as 2-oxoindole-3-acetic acid or other metabolites [41]. Although no degradation-related genes were identified in either *B. licheniformis* or *S. cerevisiae*, both of them might contribute to transformation of IAA, thereby leading to lowering extracellular concentrations. Addressing these questions will be crucial for establishing the signaling network of IAA in microbial ecology. Beyond these limitations, this study provided a framework for exploring how metabolite-based signaling shapes microbial adaptation in complex bacterial-fungal interactions.

## Conclusion

This study revealed the adaptive mechanism by which *B. licheniformis* responded to *S. cerevisiae* stress through IAA. Through multi-omics analyses, we constructed a “self-rescue” model in *B. licheniformis*, where IAA regulated its metabolic network to suppress biofilm formation and promote growth recovery. This research expands the catalog of signaling molecules in *Bacillus* and offers a novel insight into microbial adaptation strategies in *Bacillus*.

## Supplementary Material

ycag064_Supplemental_Files

## Data Availability

The transcriptomic data of *B. licheniformis* monoculture, *S. cerevisiae* monoculture, coculture, and *B. licheniformis* with the supplement of 0.25 mM IAA were submitted to the National Center for Biotechnology Information with accession number PRJNA1332830. The nontargeted metabolomic data and related analysis results were submitted to GitHub (https://github.com/SSxlei/Bl-and-Sc-interaction).
