# Supplementary material for: Indole-3-acetic acid-mediated self-rescue in Bacillus licheniformis against Saccharomyces cerevisiae stress
Source: ISME Commun. 2026 Mar 18;6(1):ycag064. doi: 10.1093/ismeco/ycag064 (PMC13098187; doi:10.1093/ismeco/ycag064)
Supplement: ycag064_Supplemental_Files [file ycag064_supplemental_files.zip › Supplementary_Materials_ycag064.docx]

Supplementary Materials for

**Indole-3-acetic acid-****mediated self-rescue in *Bacillus licheniformis* against *Saccharomyces cerevisiae* stress**

Lei Xu *et al.*

*Corresponding author. Email: [wuq@jiangnan.edu.cn](mailto:wuq@jiangnan.edu.cn)

**This file includes:**

Supplementary Fig. S1 to S11.


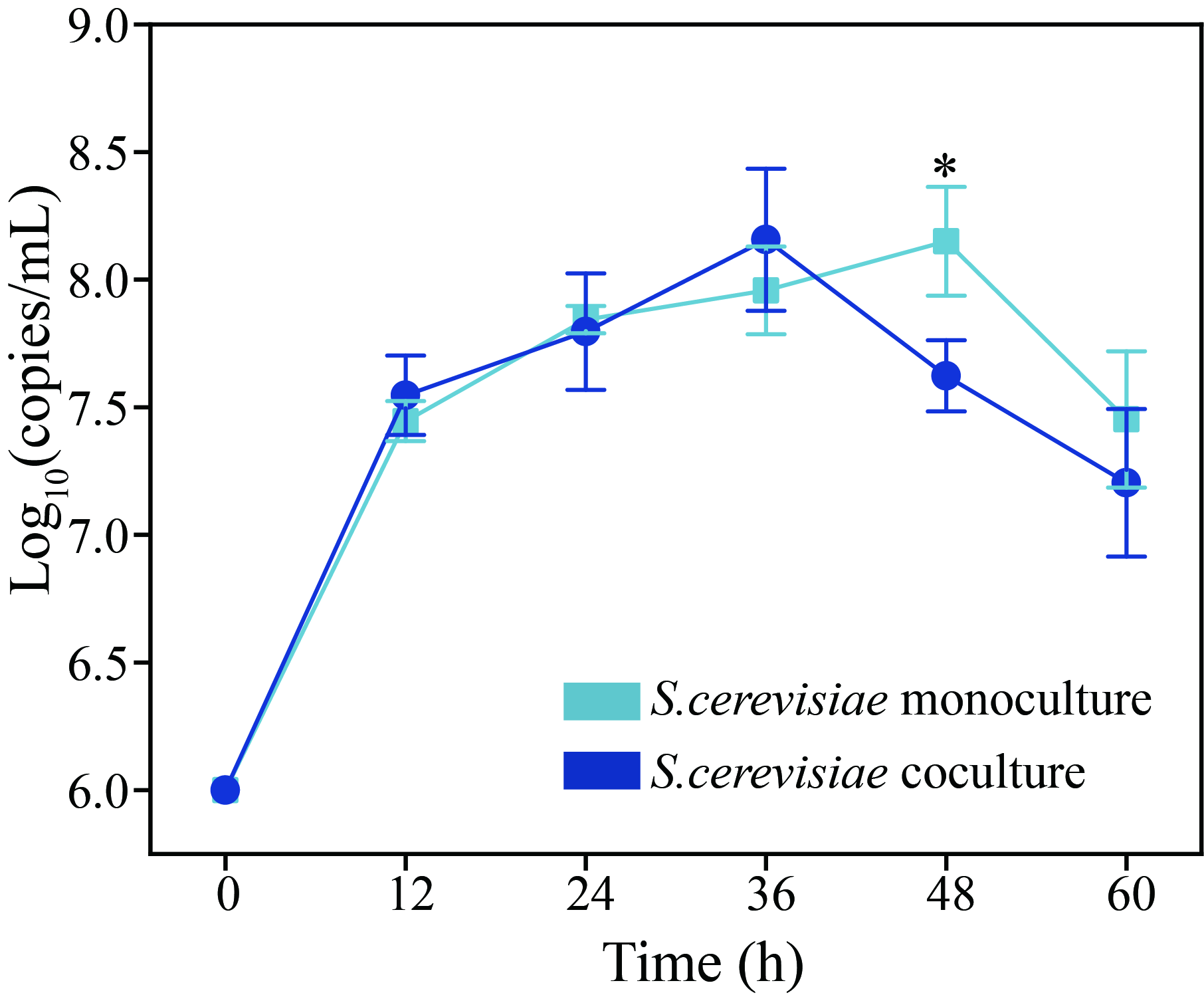


**Fig. S1 The biomass of** ***S. cerevisiae* in monoculture and coculture.** Significance: * *P* < 0.05, *t*-test.


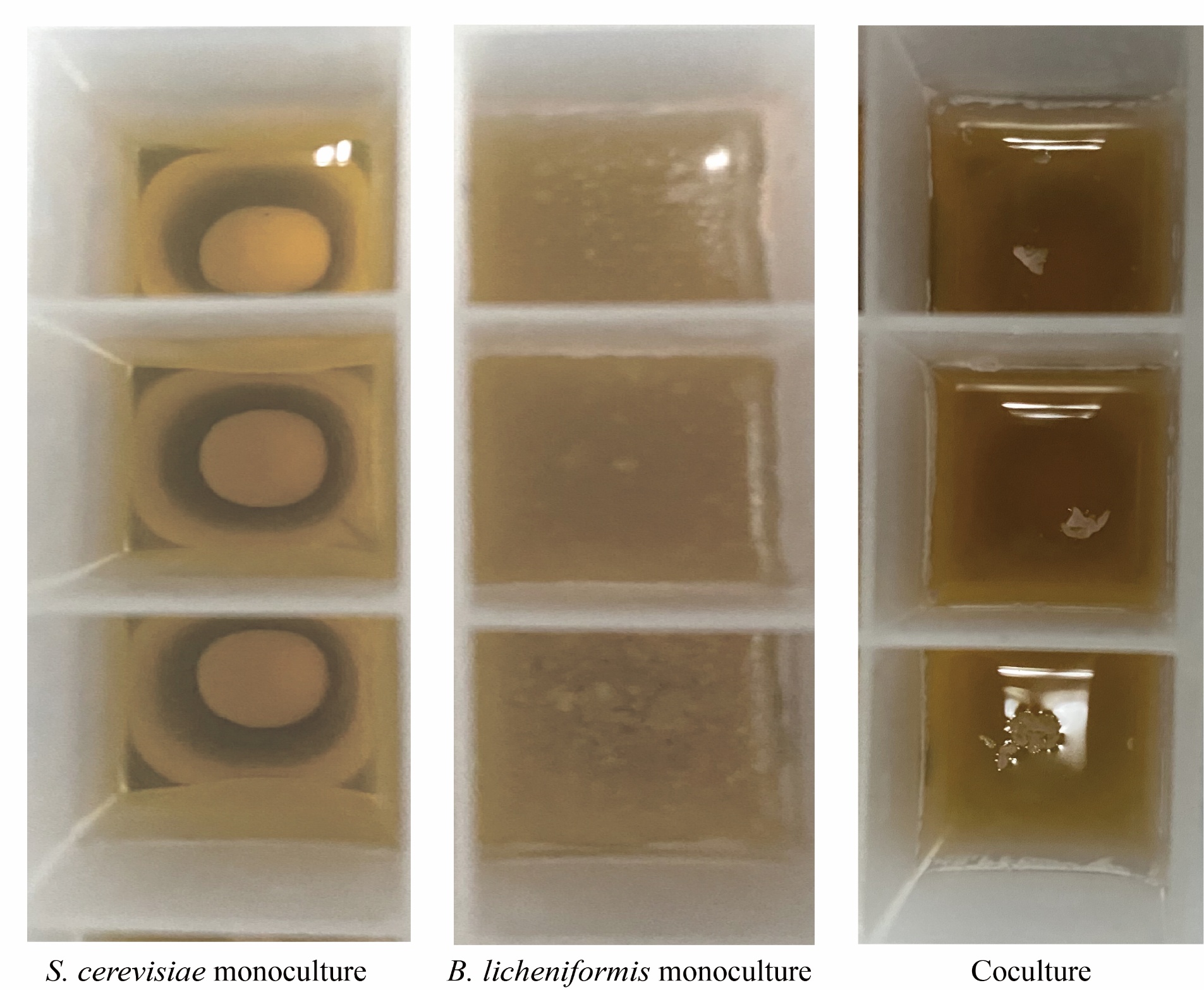


**Fig. S2 Coculture reduces biofilm formation of *B. licheniformis*.** *S. cerevisiae*, *B. licheniformis*, and coculture of *B. licheniformis* and *S. cerevisiae* were cultured separately in 24 deep-well plates.


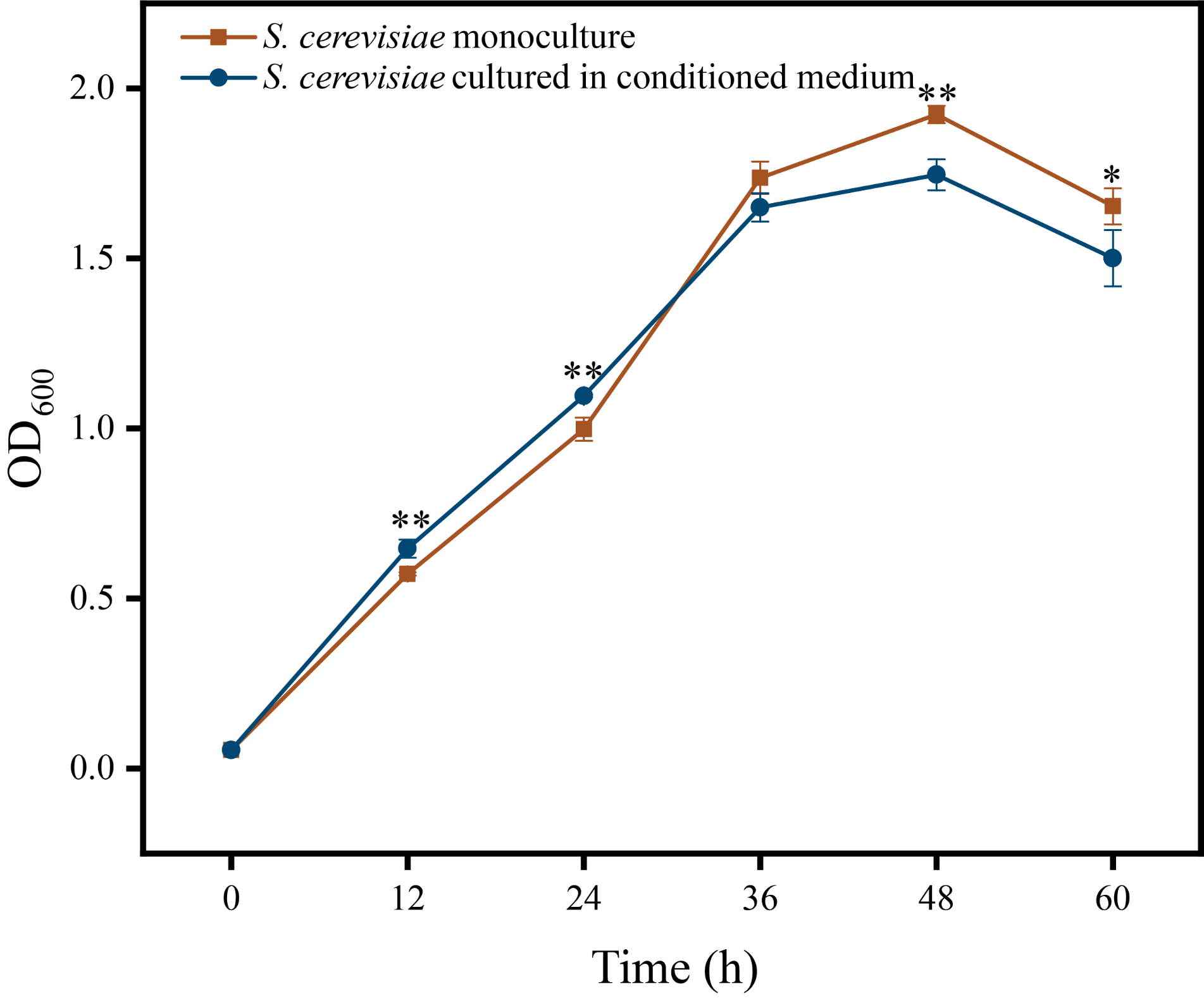


**Fig. S3 The biomass of *S. cerevisiae* in monoculture and conditioned culture.** Significance: * *P* < 0.05, ** *P* < 0.01, *t*-test.

**
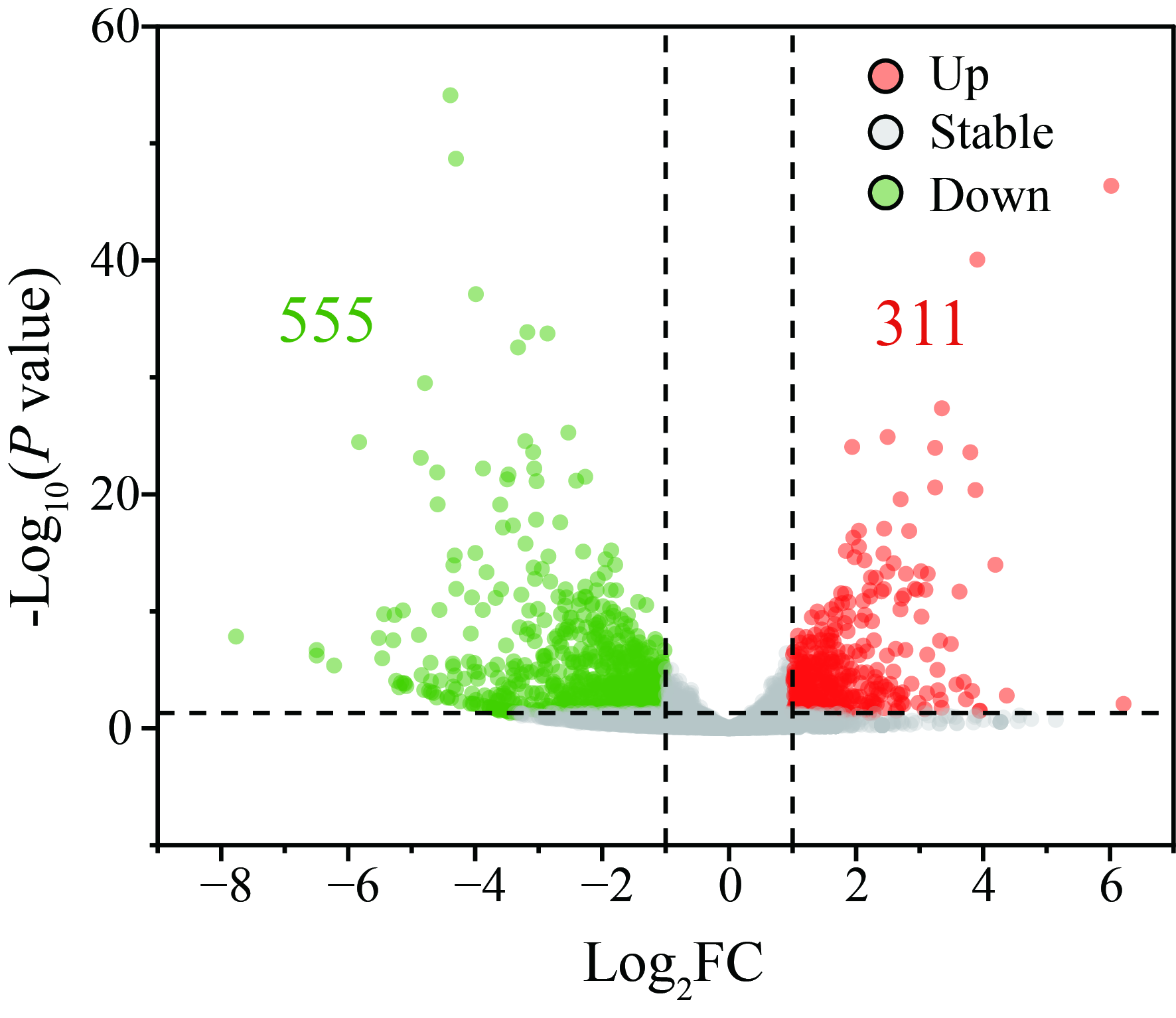
**

**Fig. S4 The transcriptomic profile of *B. licheniformis*.** Volcano diagram of differentially expressed genes (DEGs) of *B. licheniformis*. *P* value <0.05 and log_2_FC >1 were used to select significantly DEGs.

**
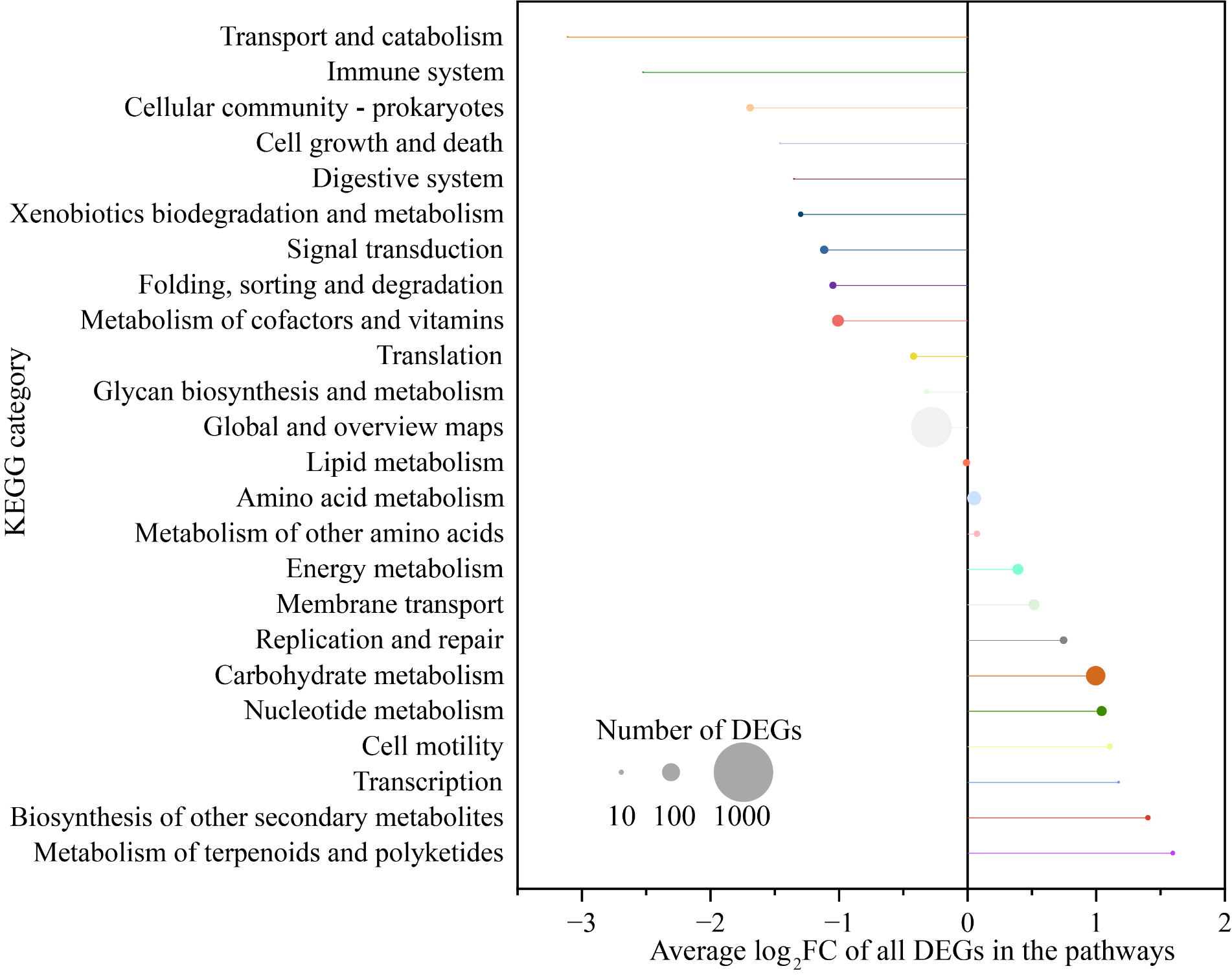
**

**Fig. S5 Kyoto encyclopedia of genes and genomes (KEGG) categories summary of differentially expressed genes (DEGs).** Bubble size indicates the number of DEGs.


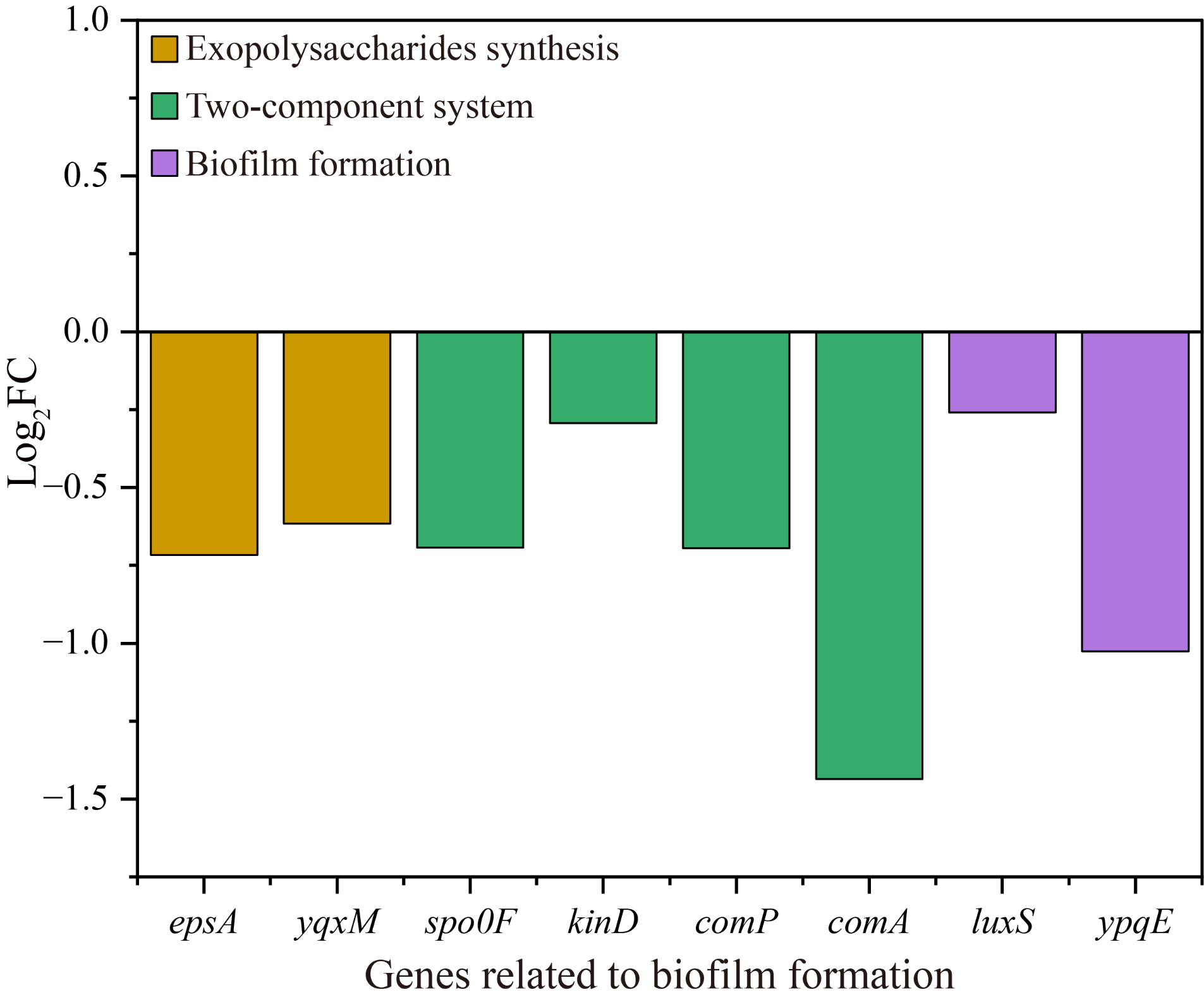


**Fig. S6 Fold changes (FC) in genes related with biofilm formation by reverse transcription qPCR.**


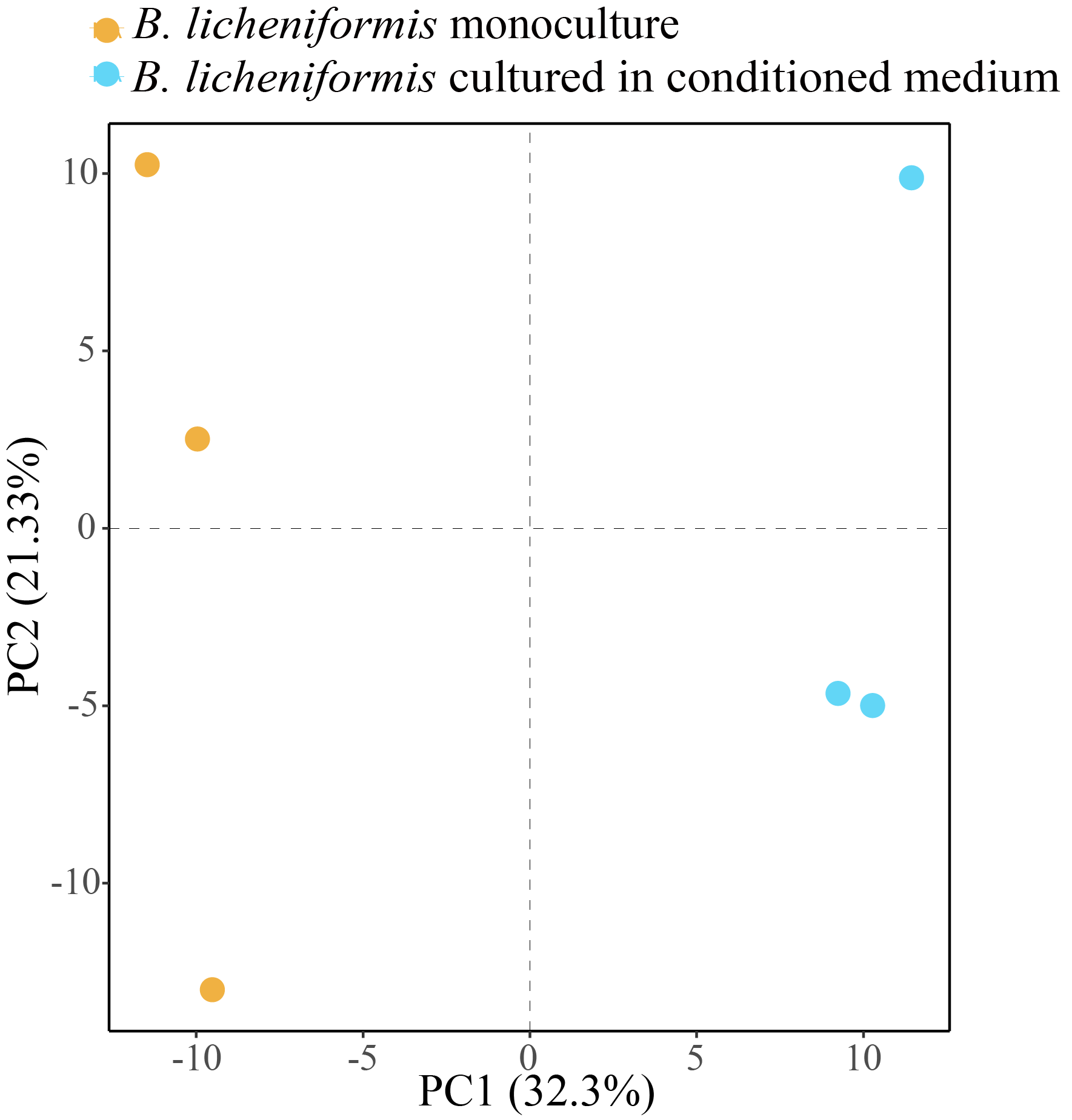


**Fig. S7 Principal component analysis (PCA) of metabolome of *B. licheniformis* in monoculture and conditioned-culture.**


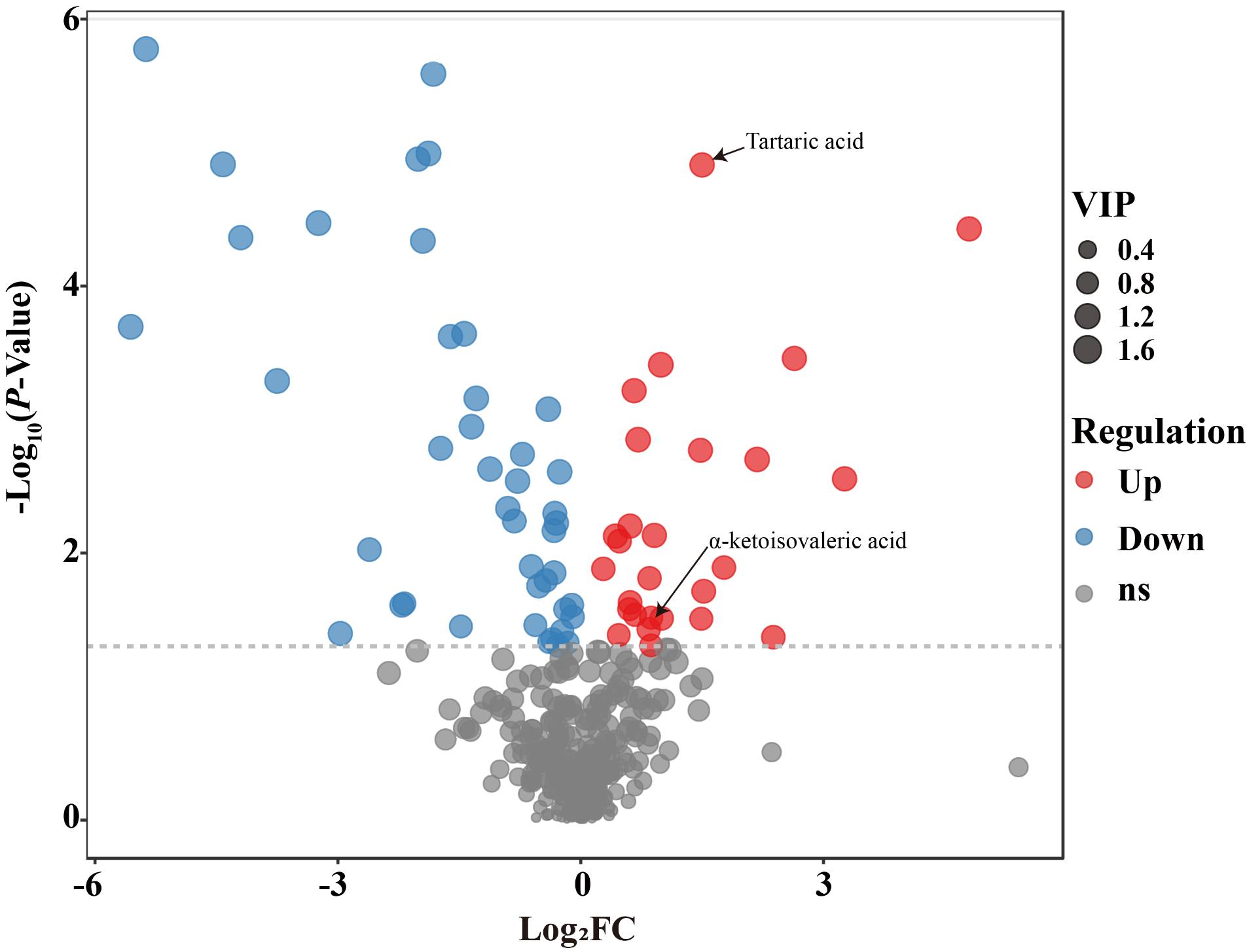


**Fig. S8 Volcano diagram of differentially abundant metabolites of *S. cerevisiae*.** *P* value <0.05 and VIP >1 were used to select differentially abundant metabolites.


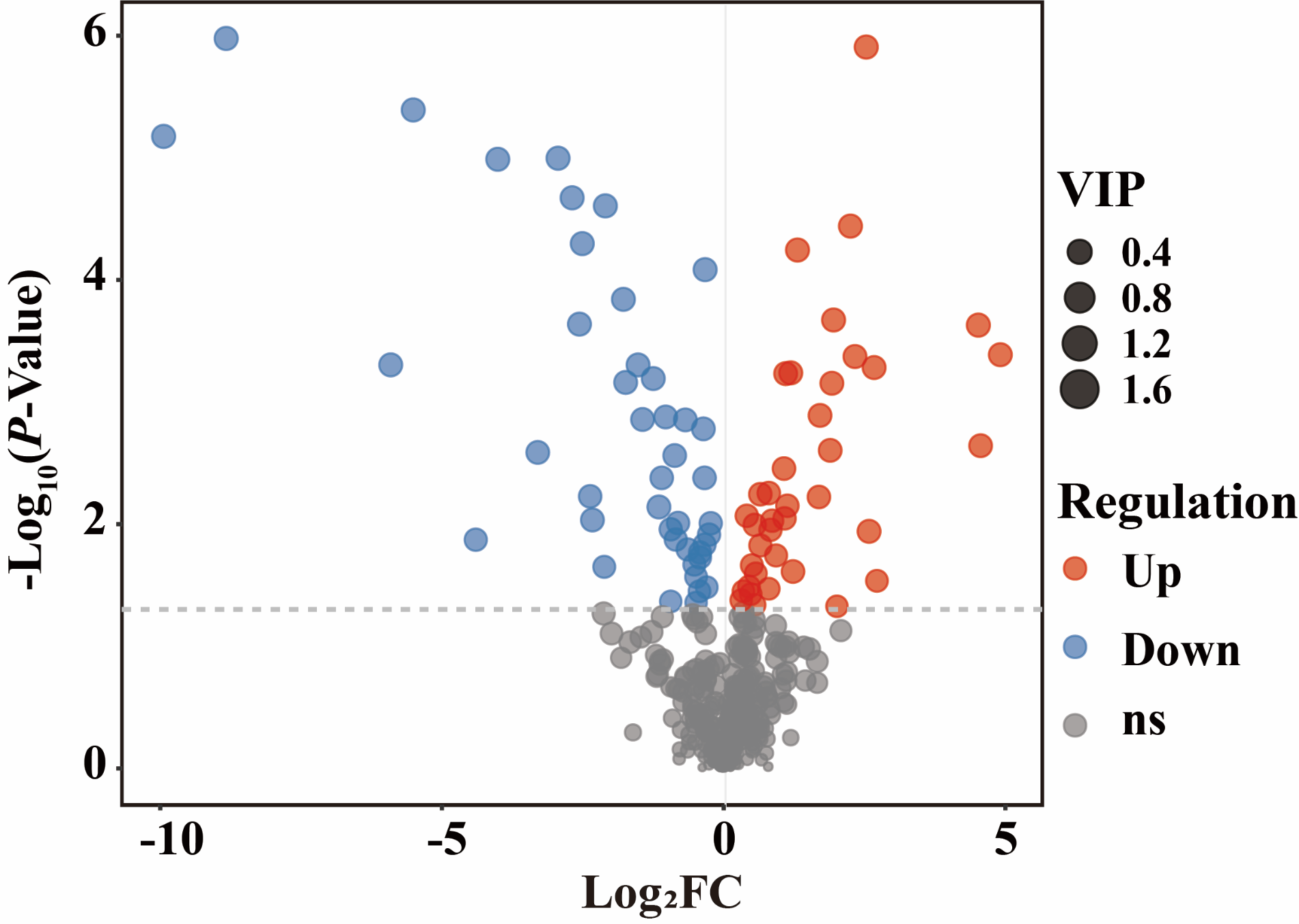


**Fig. S9 Volcano diagram of differentially abundant metabolites of *B. licheniformis***. *P* value <0.05 and VIP >1 were used to select **differentially abundant metabolites**.


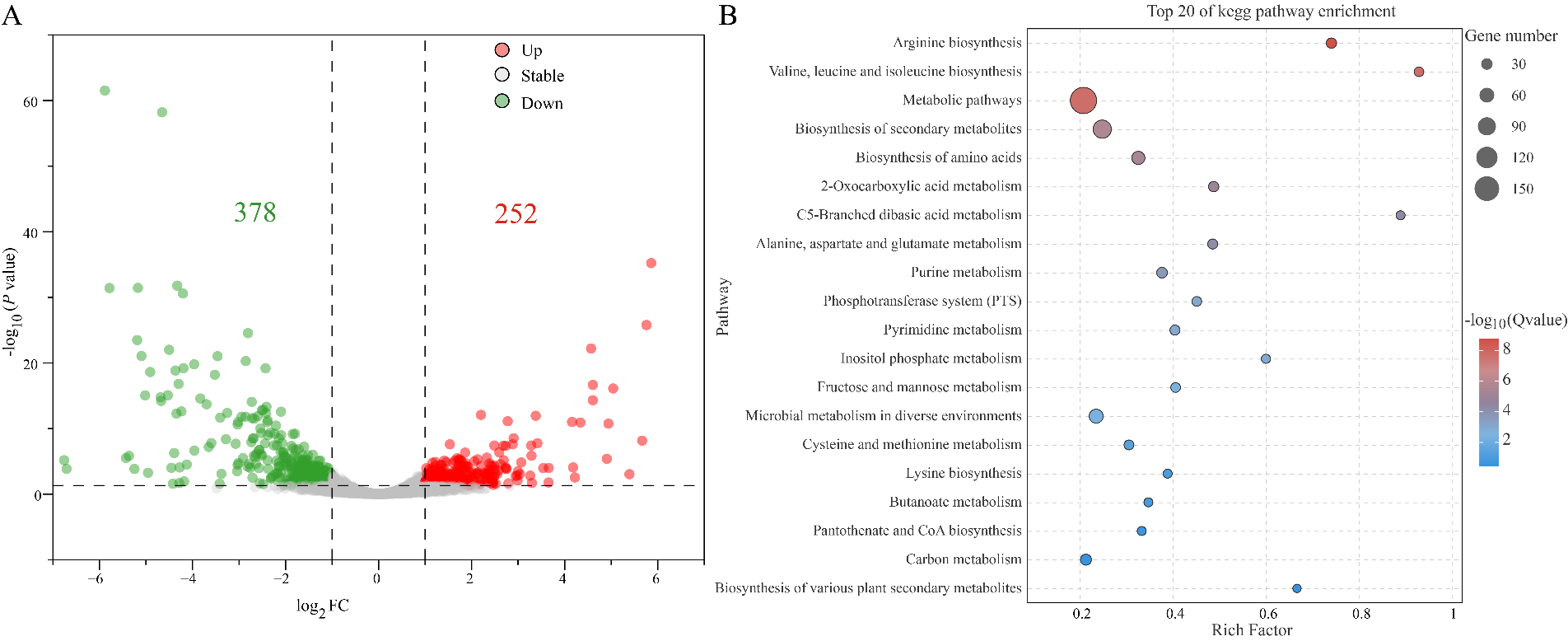


**Fig. S10 The transcriptomic profile of *B. licheniformis*** **in monoculture with 0.25 mM indole-3-acetic acid and monoculture. A** Volcano diagram of differentially expressed genes (DEGs) of *B. licheniformis*. *P* value <0.05 and log_2_(fold change) >1 were used to select significantly DEGs. **B** Top 20 of KEGG enrichment pathways of DEGs of *B. licheniformis*.


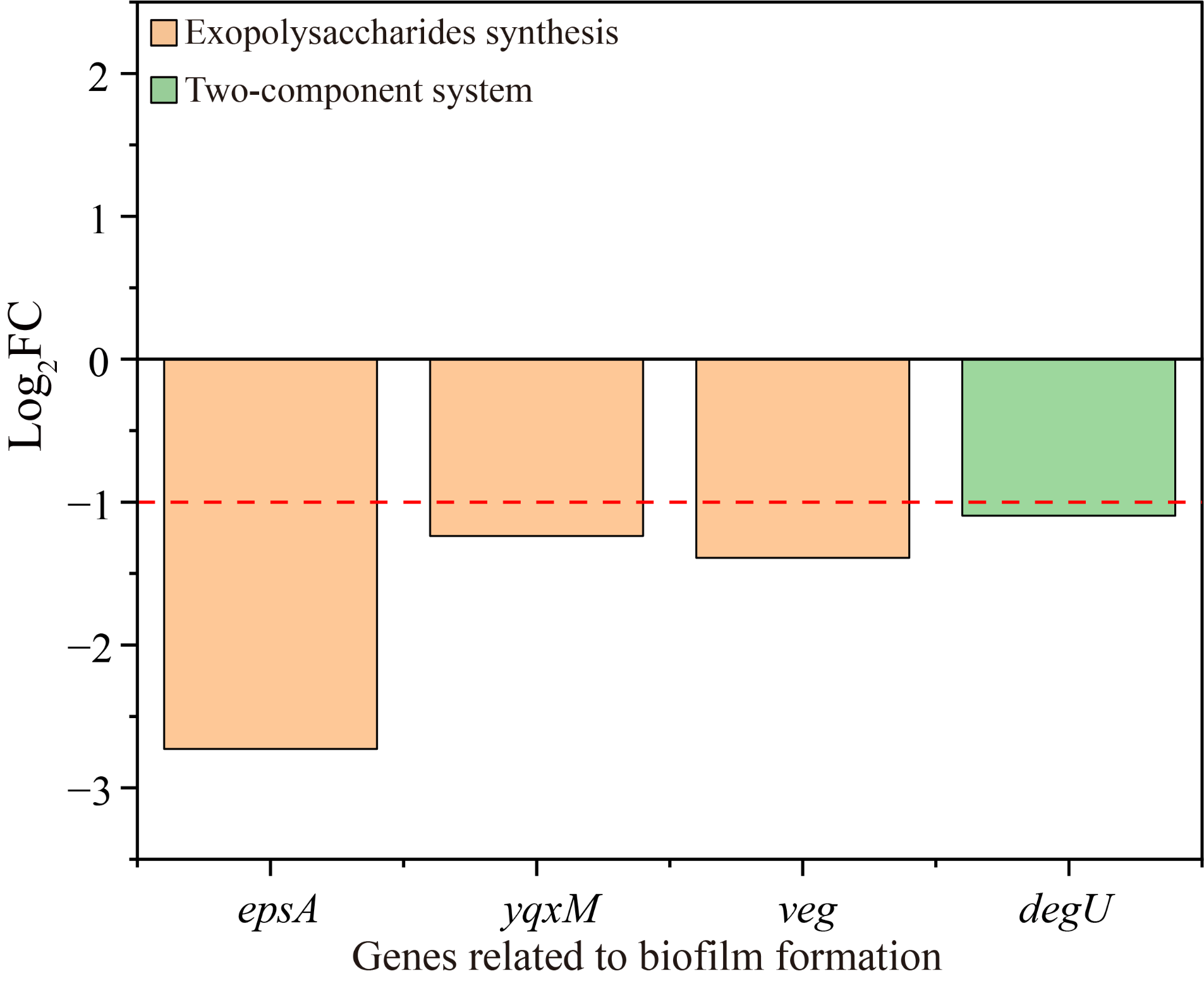


**Fig. S11 Fold changes (FC) in genes related with biofilm formation of *B. licheniformis* in monoculture with 0.25 mM indole-3-acetic acid (IAA) and monoculture.**
